# Small-molecule inhibitors of PD-1/PD-L1 immune checkpoint alleviate the PD-L1-induced exhaustion of T-cells

**DOI:** 10.18632/oncotarget.20050

**Published:** 2017-08-07

**Authors:** Lukasz Skalniak, Krzysztof M. Zak, Katarzyna Guzik, Katarzyna Magiera, Bogdan Musielak, Magdalena Pachota, Bozena Szelazek, Justyna Kocik, Przemyslaw Grudnik, Marcin Tomala, Sylwia Krzanik, Krzysztof Pyrc, Alexander Dömling, Grzegorz Dubin, Tad A. Holak

**Affiliations:** ^1^ Department of Organic Chemistry, Faculty of Chemistry, Jagiellonian University, 30-060 Krakow, Poland; ^2^ Malopolska Centre of Biotechnology, Jagiellonian University, 30-387 Krakow, Poland; ^3^ Faculty of Biochemistry, Biophysics and Biotechnology, Jagiellonian University, 30-387 Krakow, Poland; ^4^ Department of Drug Design, University of Groningen, 9713 AV Groningen, The Netherlands

**Keywords:** PD-1, PD-L1, small-molecules, immune checkpoint blockade, inhibitor

## Abstract

Antibodies targeting the PD-1/PD-L1 immune checkpoint achieved spectacular success in anticancer therapy in the recent years. In contrast, no small molecules with cellular activity have been reported so far. Here we provide evidence that small molecules are capable of alleviating the PD-1/PD-L1 immune checkpoint-mediated exhaustion of Jurkat T-lymphocytes. The two optimized small-molecule inhibitors of the PD-1/PD-L1 interaction, BMS-1001 and BMS-1166, developed by Bristol-Myers Squibb, bind to human PD-L1 and block its interaction with PD-1, when tested on isolated proteins. The compounds present low toxicity towards tested cell lines and block the interaction of soluble PD-L1 with the cell surface-expressed PD-1. As a result, BMS-1001 and BMS-1166 alleviate the inhibitory effect of the soluble PD-L1 on the T-cell receptor-mediated activation of T-lymphocytes. Moreover, the compounds were effective in attenuating the inhibitory effect of the cell surface-associated PD-L1. We also determined the X-ray structures of the complexes of BMS-1001 and BMS-1166 with PD-L1, which revealed features that may be responsible for increased potency of the compounds compared to their predecessors. Further development may lead to the design of an anticancer therapy based on the orally delivered immune checkpoint inhibition.

## INTRODUCTION

Anticancer therapies targeting immune checkpoint receptors have witnessed a spectacular success in the last years. Antibodies blocking CTLA4 interaction with CD80, and, to even more extent, PD-1/PD-L1 interaction, provided unprecedented results. This merited accelerated approvals by regulatory agencies, offering a real cure to certain formerly lethal cancers [1–5].

PD-1 is expressed on activated T cells [6] and transduces inhibitory signal which antagonizes the activating T-cell receptor (TCR) and CD28 axis [7]. The inhibitory signal is provided by PD-L1, the ligand of PD-1, which is naturally expressed on the antigen-presenting cells (APCs) and in a variety of tissues [8]. In normal conditions this mitigates T-cell response, best demonstrated in PD-L1-deficient mice where T-cell responses are markedly enhanced [9]. The immunosuppressive function of PD-L1 is utilized by cancer cells to avoid being killed by the T cells recognizing neoantigens at their surface [7, 10]. Prolonged exposure to PD-L1 leads to T cell exhaustion characterized by a sustained poor effector function [11, 12]. This is a common histological picture in which tumor tissue is infiltrated by immune cells which recognize, but are unable to eradicate the cancer cells [13, 14].

Recent data demonstrate that PD-L1 not only is overexpressed on the surface of cancer cells, but the level of soluble PD-L1 (sPD-L1) is elevated in the plasma of some cancer patients [15, 16]. It is unclear if this is related to shedding from the cancer cells or other mechanisms, but the elevated level of sPD-L1 correlates with poor prognosis [17, 18]. Of note, like the membrane-bound PD-L1, sPD-L1 has also been shown to be a negative regulator of activated T cells [17–19].

Antagonizing the PD-1/PD-L1 interaction reverts the exhausted phenotype of T cells and allows efficient killing of cancer cells [20, 21]. The utility of this approach has been demonstrated in clinics and has become a spectacular success in the recent years [1–3, 22]. In just 3 years the U.S. Food and Drug Administration (FDA) has approved two anti PD-1 antibodies: nivolumab (Opdivo, Bristol-Myers Squibb) and pembrolizumab (Keytruda, Merck), and three anti-PD-L1 antibodies, atezolizumab (Tecentriq, Genentech/Roche), durvalumab (Imfinzi, AstraZeneca) and avelumab (Bavencio, EMD Serono, Inc.), rising hope in patients suffering from cancers, which were deadly prior to the introduction of checkpoint inhibitors.

The development of small-molecular weight inhibitors is expected to bring a number of profits in the field of the PD-1/PD-L1 immune checkpoint blockade (ICB). This is due to several advantages of small molecules over therapeutic antibodies, which are: lower production costs, higher stability, improved tumor penetration, amenability for oral administration and elimination of immunogenicity issues [22]. However, development of small-molecule antagonists is lagging behind that of antibodies primarily due to the challenge of targeting a relatively flat and highly hydrophobic PD-1/PD-L1 interaction surface [23]. Only a single class of small molecules has been convincingly shown to directly antagonize this interaction [23]. These are the compounds designed and patented by Bristol-Myers Squibb (BMS) [24, 25]. We have recently demonstrated that these BMS compounds bind to human PD-L1 and block its interaction with human PD-1 [26]. Here we show that these small-molecule compounds are indeed capable of restoring the activity of T cells by disrupting the PD-1/PD-L1 interaction. We also present structural details of the interaction between PD-L1 and two improved BMS compounds, BMS-1001 and BMS-1166. This opens exciting possibilities for future development of orally bioavailable anticancer ICB therapies using small-molecule inhibitors.

## RESULTS

### Compound selection

**Figure 1:**
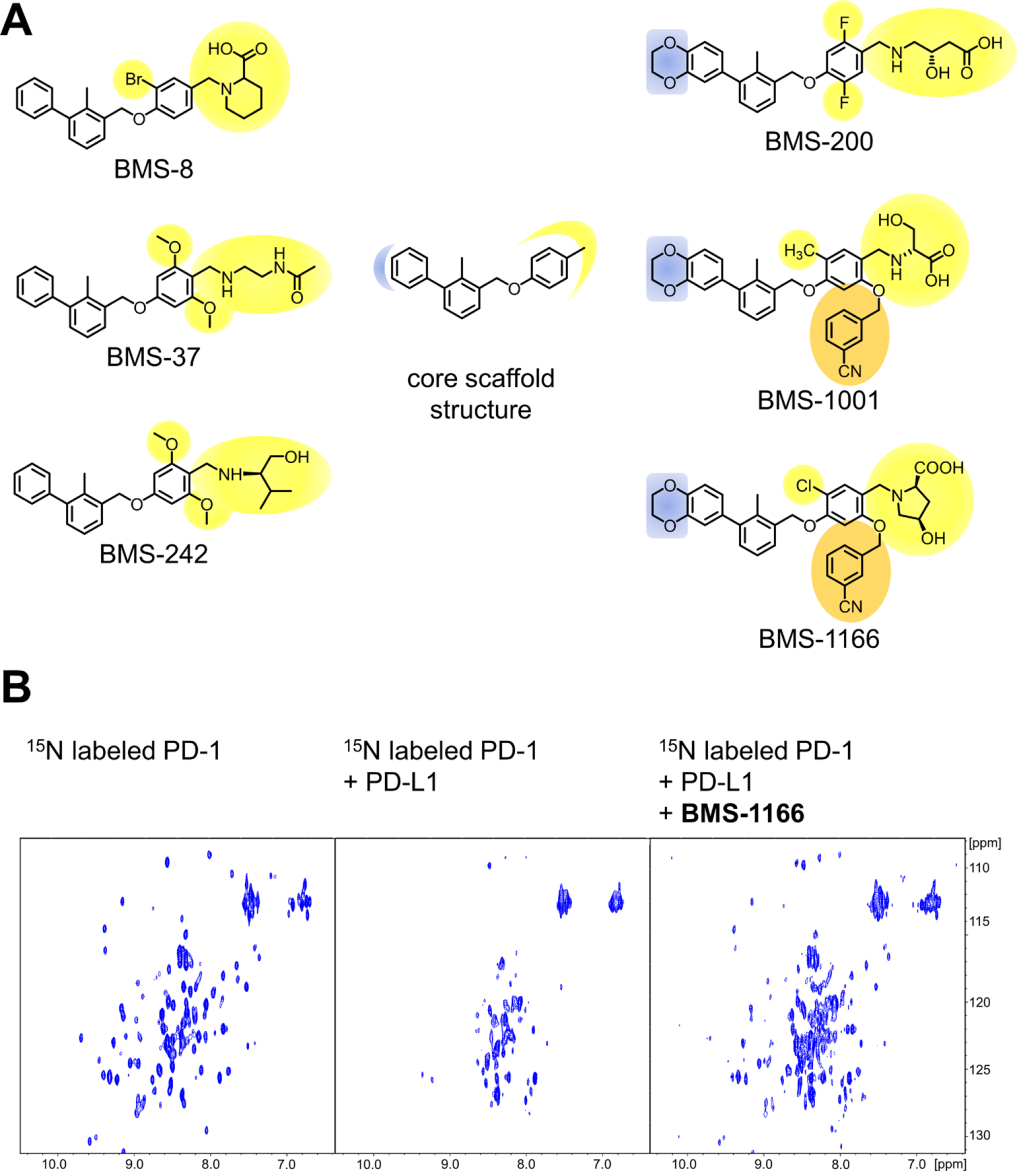
Structures and the PD-1/PD-L1 blocking potential of BMS compounds (**A**) The structures of BMS compounds tested in this study. The original numbering found in the patents [24, 25] is used. (**B**) BMS-1166 dissociates a preformed PD-1/PD-L1 complex, as shown by the NMR-based Antagonist Induced Dissociation Assay (AIDA). ^1^H-^15^N HMQC spectra are shown for the ^15^N labeled PD-1 (left) and the complex of the ^15^N labeled PD-1 and unlabeled PD-L1 alone (center) and after addition of BMS-1166 (right). Linewidth broadening, observed as loss of resonance signals in the central panel, indicates increased transverse relaxation rates associated with the complex formation. At the PD-L1:BMS-1166 molar ratio of 4:1, BMS-1166 efficiently disrupted the PD-1/PD-L1 complex, as visualized by the restoration of the ^1^H-^15^N signals of PD-1.

Based on the two recent Bristol-Myers Squibb patents [24, 25], the selection of the BMS compounds was done to obtain high diversity of the chemical structures along all modifications of distal, flexible moieties exposed to the solvent (Figure 1A). The compounds were synthesized as described previously [26] with modifications. All compounds were tested for the interaction with PD-L1 using the ^1^H-^15^N HMQC NMR titration experiment with positive results, as reported elsewhere [26] and in this manuscript (see below). In order to verify the blockade of the PD-1/PD-L1 interaction by the compounds an NMR-based AIDA was performed [27, 28]. Upon the PD-L1 binding the broadening of ^1^H-^15^N resonance signals of the ^15^N labeled PD-1 is observed due to an increased transverse relaxation rate of the higher molecular weight entities [27, 28]. This results in disappearance of the HMQC resonance signals in the spectrum (Figure 1B). All the compounds efficiently disrupted the complex formed by human PD-1 and PD-L1 proteins, as visualized by the restoration of narrow ^1^H-^15^N signals of PD-1 ([26] and (Figure 1B)).

### Analysis of unspecific toxicity of the BMS compounds

**Figure 2:**
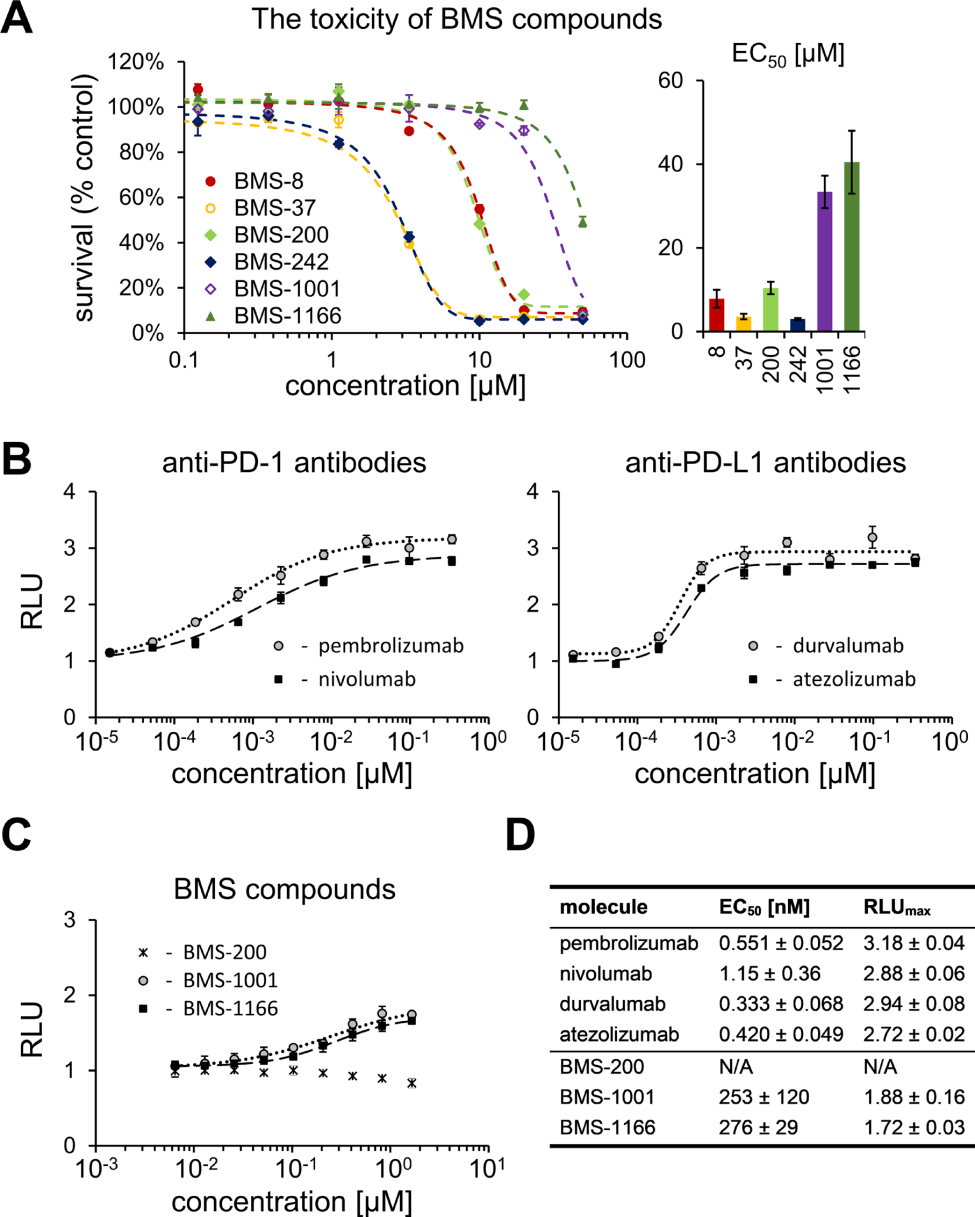
Cytotoxicity and activity of BMS compounds in PD-1/PD-L1 checkpoint assay (**A**) Cytotoxicity of BMS compounds against the PD-1 Effector Cells was tested using metabolic activity assay following the 48 h treatment of the cells with the indicated compounds. The presented EC_50_ values (right panel) are means ± SEM from the three independent experiments (left panel shows a representative result). (**B**, **C**) Activities of reference antibodies (B) and BMS compounds (C) in alleviating the effect of PD1/PD-L1 checkpoint on TCR-mediated T cell activation are expressed as the level of luciferase activity (for details see Materials and Methods). The graphs present relative luminescence normalized to the DMSO-treated controls and are representative of the three independent experiments. (**D**) The result of the fitting of Hill model to experimental data presented on panels B and C. EC_50_ values represent half maximal effective concentrations, and RLU_max_ values represent maximal relative luminescence values, and illustrate the potency of the listed molecules in restoring the activity of ECs in the assay.

To define the maximum admissible concentrations of BMS compounds for the use in the cell-based assays, the toxicity of the compounds was evaluated with the use of metabolic activity assay. For this, modified Jurkat T cells (ECs) used in all further assays were exposed to the increasing concentration of the tested compounds for 48 h. Considerable differences in the toxicity of the tested compounds were noted. The most toxic compounds (BMS-37 and −242) showed EC_50_ between 3 and 6 μM. BMS-1001 and BMS-1166 were significantly less toxic, with EC_50_ values of 33.4 and 40.5 μM. Other two compounds (BMS-8 and −200) showed moderate toxicity (Figure 2A). Low toxicity of BMS-1001 and −1166 compounds was confirmed using modified CHO-K1 cells (aAPCs; Supplementary Figure 1). These two compounds were considered the most suitable for the cell-based experiments and used as references in all further analyses.

### BMS-1001 and BMS-1166 antagonize the inhibitory effect of PD-1/PD-L1 immune checkpoint on T cell activation

A T lymphocyte-like cell line (Jurkat) modified to constitutively express PD-1 and carrying a luciferase reporter gene driven by TCR-inducible NFAT response element (Effector Cells, ECs) was used in T cell activation experiments. When antigen-presenting surrogate CHO cells, constitutively expressing the TCR agonist and PD-L1 (aAPCs) are presented to these ECs in co-culture, TCR signaling is repressed by PD-1 and the reporter remains silenced. Agents efficiently interfering with the PD-1/PD-L1 interaction activate the expression of the reporter gene resulting in the increase in luminescence intensity. Clinically relevant PD-1-antagonizing antibodies (nivolumab and pembrolizumab) and the PD-L1-blocking antibodies (atezolizumab and durvalumab) release TCR signaling with EC_50_ values in range of 0.333–1.15 nM (Figure 2B, 2D). Both BMS-1001 and BMS-1166 dose-dependently induced the activity of luciferase, demonstrating the antagonizing potential towards the PD-1/PD-L1 immune checkpoint at the cell interface (Figure 2C). Nevertheless, the effect of BMS compounds was significantly less pronounced compared to the tested antibodies, with EC_50_ values in three-digit nanomolar range and lower maximal cell activation levels, represented by lower RLU_max_ values (Figure 2D). BMS-200 did not affect the reporter expression at the concentrations below the cytotoxic level.

### BMS-1001 and BMS-1166 antagonize the inhibitory effect of soluble PD-L1 on T cells

**Figure 3:**
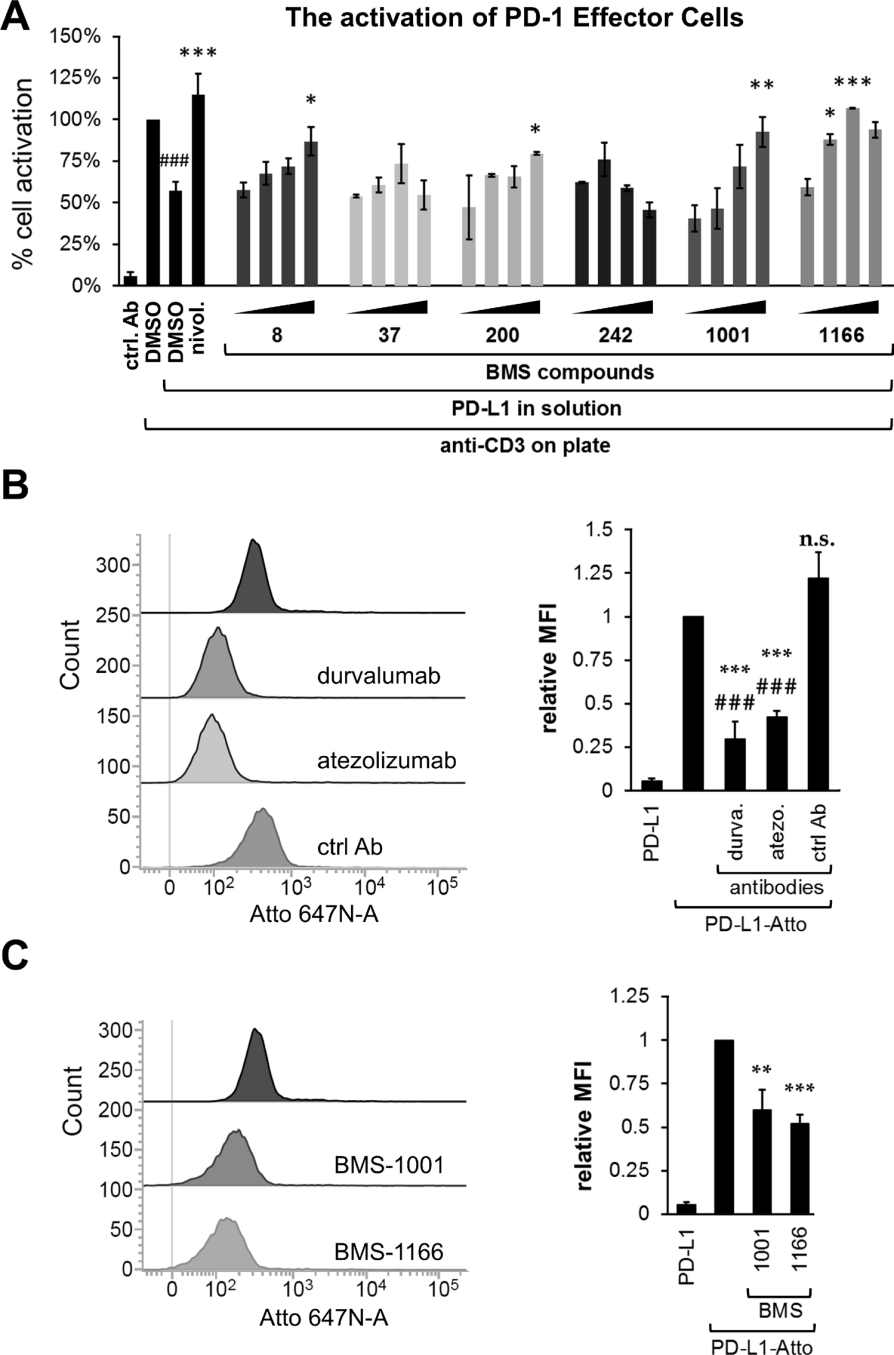
BMS compounds restore the sPD-L1-supressed activation of Jurkat T-cells (**A**) Effector Cells were activated for 24 h with the anti-CD3 antibody alone or in the presence of sPD-L1, pre-incubated with nivolumab (nivol.), DMSO or the BMS compounds. The activity of luciferase, expressed in response to TCR-mediated induction of NFAT-responsive promoter, was determined as an indicator of cell activation. BMS compounds dose-dependently restored the activation, pre-blocked by the presence of sPD-L1. The graphs present mean ± SEM from at least three independent experiments. Statistical significance was evaluated using a one-way ANOVA with the Tukey's post-hoc test: *p < 0.05, **p < 0.01, ***p < 0.001. (**B**, **C**) The binding of the fluorescently-labeled sPD-L1 (PD-L1-Atto) to PD-1 expressing cells determined using flow cytometry. PD-L1-Atto was pre-incubated with tested compounds prior to staining. Cell staining is blocked in the presence of the anti-PD-L1 antibody (durvalumab, durva. or atezolizumab, atezo.) and BMS compounds. MFI – Geo Mean Fluorescence Intensity values. The bar graphs present mean ± SEM from three independent experiments. For the statistics, *t*-test was used: **p < 0.01, ***p < 0.001.

Increased level of sPD-L1 is thought to negatively affect the T cell anticancer response [17–19]. This is because sPD-L1 provides the same inhibitory signal as the cell surface PD-L1. This is evident when ECs are simultaneously treated with anti-CD3 and sPD-L1. Anti-CD3 induced the activation of ECs, manifested in increased reporter expression (Figure 3A). Concurrent presence of sPD-L1 decreased reporter expression to about half of that observed for the anti-CD3 antibody alone (Figure 3A). The two sPD-L1 muteins, bearing mutations of the key residues at the interaction surface, PD-L1(A121Q) and PD-L1(Y56A, M115A), failed to bind to PD-1 in NMR-monitored direct titration and AIDA-NMR (Supplementary Figure 2A–2E), and did not inhibit the anti-CD3 mediated activation of ECs (Supplementary Figure 2F). This proves that the modulatory effect of sPD-L1 towards the activation of ECs is mediated specifically through the engagement of PD-1. Moreover, the anti-PD-1 antibody nivolumab abrogated the effect of sPD-L1, further supporting the involvement of the PD-1 immune checkpoint receptor (Figure 3A).

To evaluate the potential of BMS compounds in abrogating the inhibitory effect of sPD-L1 on the activation of T cells, sPD-L1 was pre-incubated with tested compounds and presented to ECs together with the anti-CD3-activating antibody. BMS-1001 and −1166 dose-dependently abolished the inhibition of ECs stimulation by sPD-L1 (Figure 3A). Importantly, at the highest concentrations (2- and 5-fold molar excess over sPD-L1) the compounds completely restored cell activation back to the level observed for anti-CD3 alone. Other tested compounds showed intermediate activities. The two most cytotoxic compounds (BMS-37 and BMS-242) presented unspecific decrease in the readout level at the highest concentrations used (Figure 3A).

To test if the observed effect of BMS compounds was directly associated with the decreased sPD-L1 recruitment to the cell surface of the PD-1-expressing cells, a His-tagged sPD-L1 was labeled with the Ni-NTA-conjugated fluorescent dye and flow cytometry analysis was performed. When ECs were contacted with labeled sPD-L1, a clear staining was observed (Figure 3B). Pre-incubation of labeled sPD-L1 with PD-L1-blocking antibody durvalumab or atezolizumab significantly decreased the staining of ECs contrary to the control, non-specific antibody (Figure 3B). Additionally, the two PD-L1 mutants, PD-L1(A121Q) and PD-L1(Y56A/M115A), demonstrated reduced binding to ECs as evidenced by weaker cell staining compared to PD-L1(wt) (Supplementary Figure 2G). This demonstrates that the labeled sPD-L1 utilizes the canonical interaction surface to bind PD-1 receptor. Pre-incubation of sPD-L1 with BMS-1001 or BMS-1166 significantly decreased the intensity of staining (Figure 3B), again demonstrating that both compounds interfere with sPD-L1 interaction with PD-1 receptor exposed at the cell surface.

### Structural basis of the interaction of BMS-1001 and BMS-1166 with hPD-L1

**Figure 4:**
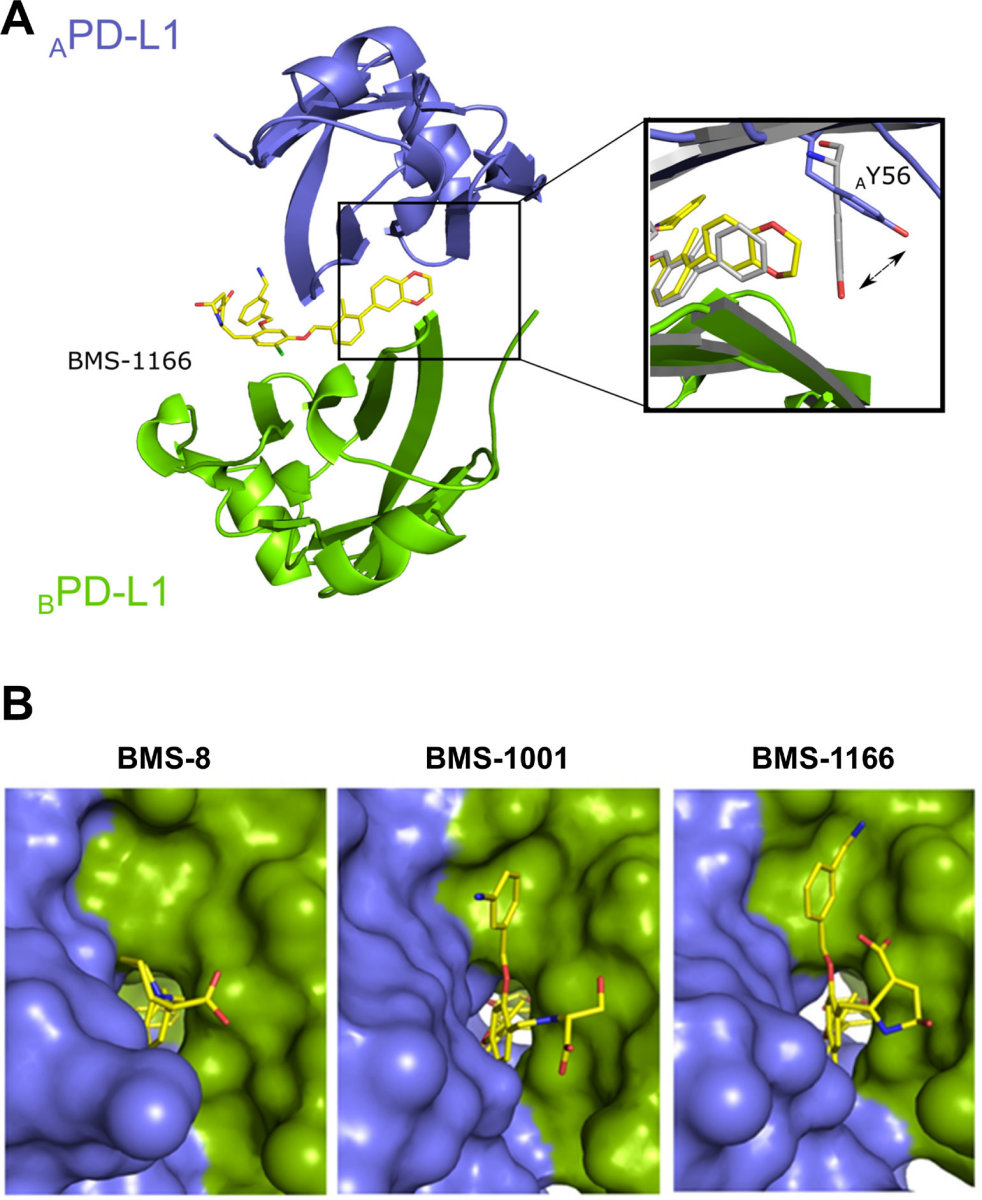
BMS-1166 induces binding cleft opening (**A**) Arrangement of the molecules in the crystal structure – two PD-L1 molecules form a single pocket accommodating BMS compound. A close-up presents the position of the side-chain of the Tyr56 in BMS-8 (gray) and BMS-1166 (yellow/blue) containing structures. (**B**) The 2,3-dihydro-1,4-benzodioxine moiety of BMS-1001 and BMS-1166 induces the previously absent side-chain movement that triggers transformation of the binding pocket into the binding tunnel across the transverse vertical axis of the dimer. The panel visualizes surface representation of the dimer with the visible binding cleft (left, BMS-8/PD-L1 complex) and the binding tunnel (center and right for the BMS-1001/PD-L1 and BMS-1166/PD-L1 complexes, respectively). PD-L1 molecules forming the dimer are colored blue and green for chains A and B, respectively. Compounds are shown as yellow sticks.

To decipher the structural details of the interaction of improved BMS compounds with PD-L1, we crystallized and solved the structure of BMS-1166 in complex with the Ig-like V-type domain of human PD-L1 at the resolution of 2.2 Å (Table 1). The asymmetric unit contains four protein molecules organized in two dimers, and each dimer harbors a single inhibitor molecule in a cylindrical tunnel at the interface of two monomers. Such organization of the dimer is similar to that we previously observed for BMS-202 [26]. The deep hydrophobic pocket harboring BMS-202 is transformed into a tunnel in the PD-L1/BMS-1166 structure by rotation of the _A_Tyr56 sidechain (the monomer molecules are annotated by subscripts A, B according to their chain arrangement in the crystal structure of the dimer) by 40 degrees (Figure 4). Not only this removes the steric hindrance, but provides additional interactions between _A_Tyr56 and the 2,3-dihydro-1,4-benzodioxine moiety of the inhibitor.

**Table 1:** Data collection and refinement statistics (molecular replacement)

	BMS-1001	BMS-1166
Data collection		
Wavelength (Å)	0.9795	0. 91842
Resolution range	46.01–2.01 (2.09–2.01)	45.94–2.20 (2.28–2.20)
Space group	P 21 21 21	P 21 21 21
Cell dimensions		
*a*, *b*, *c* (Å)	39.88 84.67 164.41	40.53 84.61 164.12
α, β, γ (°)	90 90 90	90 90 90
*R*_merge_	0.065 (0.452)	0.050 (0.240)
*I* / σ*I*	22.3 (5.3)	20 (6.3)
Completeness (%)	99.8 (97.7)	100 (100)
Redundancy	12.7 (12.7)	6.3 (6.8)
Refinement		
Resolution (Å)	2.01	2.2
No. reflections	37778 (3648)	29563 (2925)
*R*_work_ / *R*_free_	0.209/0.262	0.213/0.2563
No. atoms	4359	4210
Protein	3945	3862
Ligand/ion	96	100
Ramachandran favored (%)	97	97
Ramachandran allowed (%)	3	3
Ramachandran outliers (%)	0	0
*B*-factors		
Protein	43.91	46.14
Ligand/ion	53.40	56.19
Water	47.11	44.62
R.m.s. deviations		
Bond lengths (Å)	0.017	0.023
Bond angles (°)	1.9	1.67

*Values in parentheses are for the highest-resolution shell.

Induced pockets are attractive targets for inhibitor design. To confirm the binding mode and vulnerability of the Tyr56 sidechain to ligand-induced changes, we solved the crystal structure of BMS-1001 in complex with sPD-L1 (Table 1; Supplementary Figure 3). The arrangement of _A_Tyr56 sidechain and a resulting tunnel shaped binding pocket are identical in both structures (Figure 4).

At the other side of the tunnel the 3-cyanobenzyl substituent of BMS-1166 is stabilized through the ring π-stacking with _B_Tyr123. Additionally, hydrogen bonds are formed between the cyan group and Nε atom of the side chain of _B_Arg125. The (2*R*, 4*R*)-4-hydroxypyrrolidine-2-carboxylic acid substituent is stabilized through hydrogen bond with NH_2_ group of _B_Lys124 side chain (Figure 4 and Supplementary Figure 4). In BMS-1001, the (2*R*)-2-amino-3-hydroxypropanoic acid moiety forms hydrogen bonds with the carbonyl of _A_Asp122 sidechain and additional water-mediated hydrogen bond with the NH_2_ group of _A_Lys124 and mainchain carbonyl of _A_Tyr123. The 3-cyanobenzyl substituent provides additional hydrophobic contacts with _A_Tyr123 aromatic ring and _A_Arg125 sidechain (Figure 4 and Supplementary Figure 4).

### Defining the minimal BMS-1166 fragment with the PD-L1 binding ability

**Figure 5:**
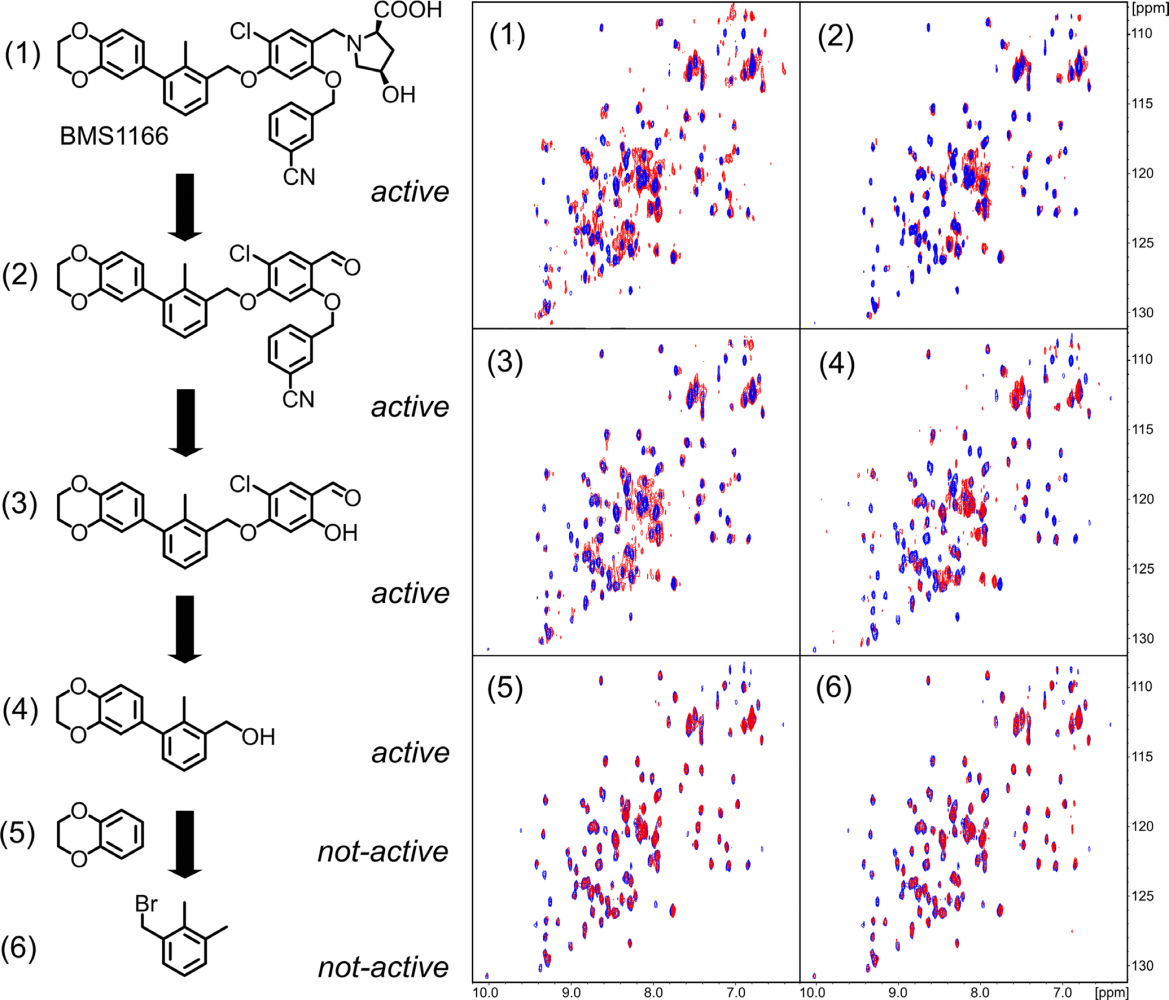
Decomposition of BMS-1166 BMS-1166 (1) or its fragments (2-6) were tested for the interaction with PD-L1 (blue) using ^1^H-^15^N HMQC NMR method. The spectra determined in presence of compounds (1)-(4) display line-broadening (red), indicative of compound-induced formation of a higher molecular weight PD-L1 complex. Compounds (5) and (6) induced no changes in the spectra even at molar excess, indicating the lack of interaction.

For further compound development it is of high importance to define the minimal fragments essential for the binding to the target protein. For this, BMS-1166 and its fragments were tested for interaction with PD-L1 using NMR method. From the six decomposition fragments tested, four retained PD-L1 binding potential (Figure 5). The analysis revealed that a two aromatic ring system (compound 4, Figure 5) is the minimal fragment of BMS-1166 responsible for the PD-L1 binding.

### BMS-1001 and BMS-1166 induce PD-L1 dimerization in solution

The formation of solution-stable PD-L1 dimers in the presence of BMS compounds was characterized previously for BMS-202 [26]. The crystal structures of BMS-1001 and −1166 bound to PD-L1 indicated similar properties of the compounds, but this required verification by in-solution assays. Initial clues were provided by direct NMR titration of ^15^N labeled PD-L1 where both BMS-1001 and BMS-1166 induced linewidth broadening indicative of significant increase in molecular weight of the analyzed species upon compound addition (Figure 5). This was verified by a crosslinking experiment. sPD-L1 preincubated in the presence of a cross-linking agent (bis(sulfosuccinimidyl)suberate, BS3) migrated in SDS-PAGE as a single band corresponding to monomeric protein. Preincubation in the same conditions with the addition of BMS-1001 resulted in two bands in SDS-PAGE analysis, corresponding to sPD-L1 monomer and a dimer (Supplementary Figure 5A). sPD-L1 dimerization in the presence of BMS was further confirmed by a size exclusion chromatography, where the retention time of sPD-L1 was significantly shortened in the presence of BMS-1001 and the change in retention time was indicative of dimer formation (Supplementary Figure 5B).

### Model of BMS-induced dimerization of PD-L1

**Figure 6:**
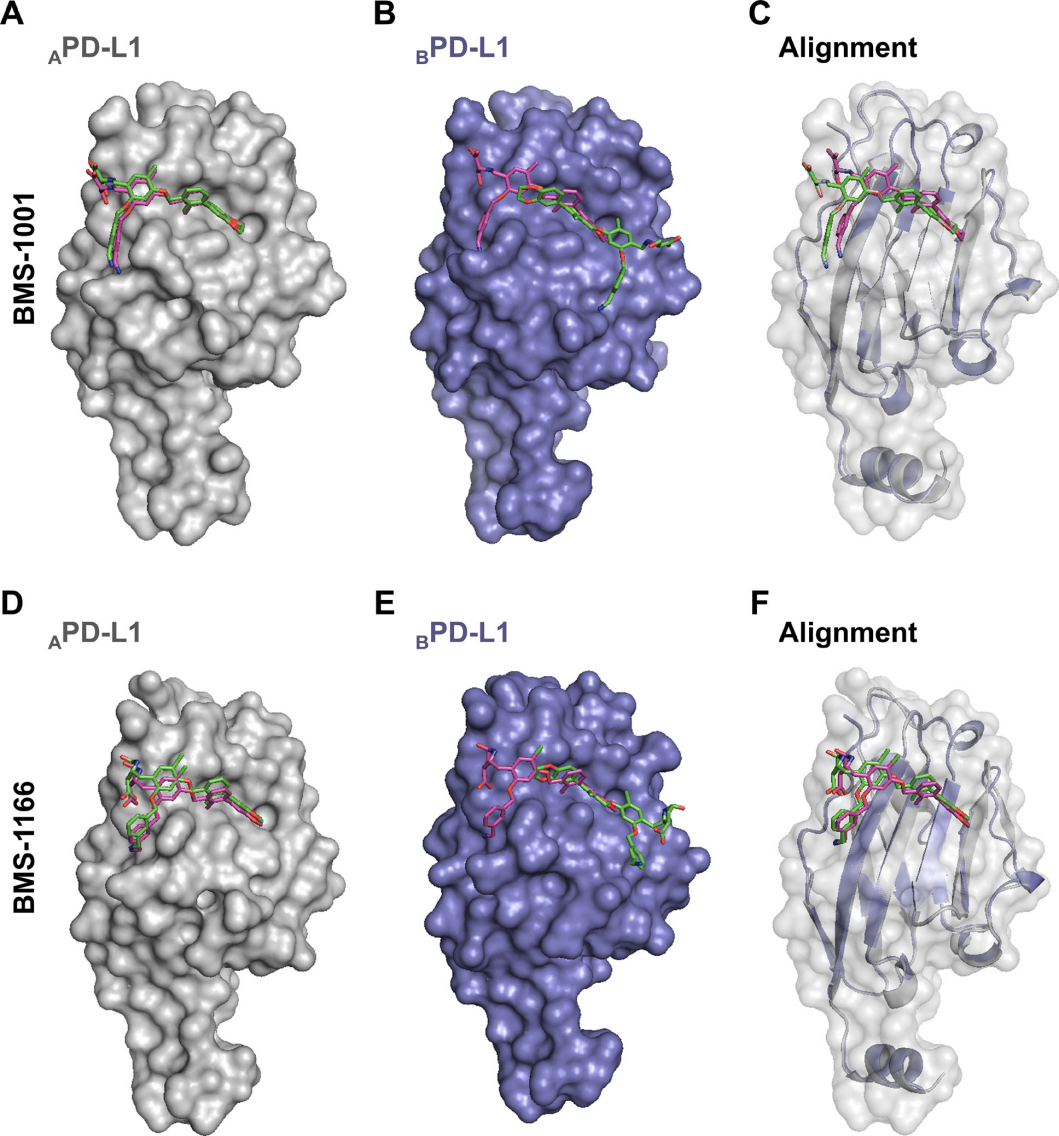
The prediction of BMS-1001 and −1166 binding sites on PD-L1 surface Molecular docking simulations were performed to predict the favored binding sites of BMS compounds to PD-L1 protein. _A_PD-L1 and _B_PD-L1 protomers were extracted from the co-crystal structures of BMS-1001/PD-L1 and BMS-1166/PD-L1 and used as templates (receptors) for the docking of appropriate BMS compound (ligand). The best docking results (represented by the purple compounds) are compared with crystallographic arrangements (represented by the green compounds). (**A**, **D**) For both BMS compounds, when _A_PD-L1 protomer was investigated, the preferred molecular docking results corresponded well to the binding modes determined from crystallographic data. (**B**, **E**) When _B_PD-L1 protomer was selected, molecular docking simulations suggested an opposite orientation of the compounds to the one defined by crystal structure analysis (among the 10 best results not a single resembled the BMS orientation determined from crystal structures of BMS/_B_PD-L1). (**C**, **F**) The alignment of _B_PD-L1 protomer to the _A_PD-L1 protomer revealed a significant resemblance of the binding mode of BMS compounds docked to _B_PD-L1 and the ones revealed from the crystals for _A_PD-L1 protomer.

Our structural and biochemical results suggest that BMS compounds induce the formation of PD-L1 dimers. Inside the dimer a single BMS molecule interacts with two distinct sites on PD-L1, within PD-1 interaction surfaces of both protomers. Further optimization of BMS compounds, especially towards the molecules which action would not require the engagement of two PD-L1 entities, requires defining the energetically favorable binding mode of BMS at the PD-L1 surface. Docking simulations of BMS-1001 and −1166 at separate protomers consistently show a single energetically favorable binding mode identical to that observed in the crystal structure of _A_PD-L1, but not _B_PD-L1 (Figure 6). This suggests a model where BMS compounds transiently bind PD-L1 in a mode characteristic for _A_PD-L1 protomer, and such a preformed complex recruits the second PD-L1 molecule. Such model, if confirmed experimentally, would direct the development of BMS compound towards monomeric binders.

## DISCUSSION

According to recent reports and clinical data, targeting the PD-1/PD-L1 immune checkpoint is an important and effective strategy for the treatment of diverse cancer types [1–3]. The use of monoclonal antibodies have evidenced the therapeutic potential of PD-1/PD-L1 blockade, but further development of clinical strategies targeting this immune checkpoint would be much facilitated by the introduction of other molecule types, devoid of the known drawbacks of monoclonal antibodies.

Several classes of non-antibody molecules have been patented in the recent years, including one group of small-molecule inhibitors, disclosed by Bristol-Myers Squibb in the two consecutive patents [24, 25], but very little data on activity was provided. In order to verify the activity of these disclosed groups of small molecules, we have selected and synthesized six representative compounds with one of the best K_d_ values measured by Homogenous Time-Resolved Fluorescence (HTRF) *in vitro* binding assay [24, 25]. Our study shows the biological activity of some of these small molecules at the cellular level and provides the background for their evaluation in further pre-clinical studies. At the same time issues and limitations of these particular compounds are highlighted, necessitating further improvement.

We have shown previously that the compounds BMS-8, BMS-37 and BMS-242 bind to PD-L1 and efficiently dissociate the human PD-1/PD-L1 complex *in vitro* [26]. Here we demonstrate that the two optimized BMS compounds, BMS-1001 and BMS-1166, present significantly improved cytotoxic properties, allowing the use of higher concentrations. In addition, unlike the three compounds described earlier, BMS-1001 and −1166 present the potential of restoring the activation of effector Jurkat T cells, attenuated by both soluble and membrane-bound PD-L1 presented by antigen-presenting cells. Although the potential of the compounds in restoring the activation of effector cells is significantly lower than that observed for the therapeutic antibodies, further optimization based on our structural data may lead to the development of more potent molecules.

Both structural and biochemical data suggest the formation of dimers of sPD-L1 in the presence of BMS compounds. This unique formation of PD-L1 dimers may facilitate the inhibition of PD-1/PD-L1 interaction, as the dimerization engages PD-1-binding surfaces of both PD-L1 protomers. Since the level of soluble PD-L1 is increased in the serum of cancer patients and correlates with poor prognosis [15, 16], and sPD-L1 is able to interfere with the activation of blood T cells [17–19], the functional elimination of sPD-L1 could provide a positive therapeutic role by increasing the immune competence of T cells circulating in the blood. Additionally, this might have a meaning in cancer lesions, where cancer-derived sPD-L1 would block membrane-bound PD-L1 molecules in the presence of the compounds evaluated in this study, or membrane-bound PD-L1 molecules would reciprocally block each other at the cell-cell interface of cancer cells. Nevertheless, these potential mechanisms require further experimental verification.

Our structural results show that the tested BMS compounds force the conformational changes at the surface of PD-L1 molecule upon binding. Both conformational changes and additional interaction sub-sites absent in previously published structures result in improved interaction between the characterized compounds and the target protein (Figure 4). These results prove that although the targeting of PD-1/PD-L1 interaction with small molecules was considered challenging due to relatively flat interface of PD-1/PD-L1 interaction, it is not unfeasible. The plasticity of PD-L1 surface seems to be instrumental in designing the new compounds. The structures of BMS compounds reported here and in our previous studies [26] and the structure of PD-1/PD-L1 complex [29] may serve as starting points for the design of new molecular scaffolds based on molecular docking strategies. On top of that, our binding model suggests the more favorable BMS binding pose to start with.

Collectively, our results advocate for PD-1/PD-L1-blocking potential of the evaluated BMS compounds and present a likely mode of this inhibition by forcing the dimerization of PD-L1 molecules.

## MATERIALS AND METHODS

### Therapeutic antibodies

Following antibodies were used: anti-PD-1 antibody nivolumab (MDX-1106, trade name: Opdivo, Bristol-Myers Squibb), anti-PD-1 antibody pembrolizumab (MK-3475, trade name: Keytruda, Merck & Co., Inc.), anti-PD-L1 antibody atezolizumab (MPDL3280A, trade name: Tecentriq, Genentech/Roche) and anti-PD-L1 antibody durvalumab (MEDI4736, trade name: Imfinzi, AstraZeneca Pharmaceuticals LP).

### Synthesis of Bristol-Myers Squibb compounds

Six small-molecule compounds disclosed by Bristol-Myers Squibb were tested in respect to their biological activities in disrupting of the PD-1/PD-L1 interaction. The compounds were synthesized according to procedures described in BMS patents [24, 25] with minor modifications as described elsewhere [26]. The identity and purity of all compounds was evaluated by ^1^H NMR, ^13^C NMR, high-resolution mass spectrometry (HRMS) and UPLC/MS.

### Protein expression and purification

Expression and purification of human PD-L1 (residues 18–134, C-terminal His-tag) and human PD-1 (residues 33–150, Cys93 exchanged to serine) was carried out as described previously [26]. A single residue A121Q hPD-L1 mutant and double mutant Y56A, M115A were prepared using site-directed mutagenesis. The constructs were verified by sequencing. The mutants were purified using the same protocol as the wild-type protein. The purity of the proteins was evaluated by SDS-PAGE and folding was examined using NMR spectroscopy.

### NMR measurements

Uniform ^15^N labelling was obtained by expressing proteins in the M9 minimal medium containing ^15^NH_4_Cl as the sole nitrogen source. For NMR measurements the buffer was exchanged by gel filtration to PBS pH 7.4 (PD-L1) or 25 mM sodium phosphate containing 100 mM NaCl pH 6.4 (PD-1). 10% (v/v) of D_2_O was added to the samples to provide the lock signal. All spectra were recorded at 300K using a Bruker Avance III 600 MHz spectrometer. The interaction of the compounds with PD-L1 was evaluated by monitoring the perturbations in chemical shifts of NMR resonances in the ^1^H-^15^N 2D HMQC upon titration with the compound. The ability of tested compounds to dissociate PD-L1/PD-1 was evaluated using the Antagonist Induced Dissociation Assay (AIDA) [27, 28]. In brief, ^15^N-labeled PD-1 (0.2 mM) was slightly overtitrated with the unlabeled PD-L1. The compounds were aliquoted into the resulting mixture. During the experiment the ^1^H-^15^N signals were monitored by the HMQC.

### Cell lines

To verify the potency of BMS compounds in the inhibition of the PD-1/PD-L1 interactions, a cell-based model of the PD-1/PD-L1 immune checkpoint blockade was used. In the assay, two model cell lines are utilized: the artificial Antigen-Presenting Cells (PD-L1^+^ aAPC/CHO-K1 cells, called aAPCs) overexpressing TCR ligand and PD-L1, and T cell surrogate, a modified Jurkat T cell line overexpressing PD-1 and carrying a luciferase reporter under the control of NFAT promoter (PD-1 Effector Cells, called ECs) [30]. The cells were obtained from Promega and cultured in the RPMI 1640 medium (Lonza) supplemented with 10% Fetal Bovine Serum (BioWest), 100 U/ml Penicillin and 100 U/ml Streptomycin. Additionally, the cells were propagated in a constant presence of Hygromycin B (50 μg/ml) and G418 (250 μg/ml) to provide a stable expression of the introduced genetic constructs. The two latter antibiotics were omitted in the experiments. Overexpression of PD-1 on ECs and PD-L1 on aAPCs was verified by flow cytometry (not shown) and the presence of the luciferase-expressing gene was verified by monitoring luciferase activity following anti-CD3 antibody stimulation. Antibiotic selection, flow cytometry and reporter expression served as cell line authentication method. The cells were periodically tested and found negative for *Mycoplasma* contamination using PCR-based method [31].

### Cytotoxicity assay

5 000 ECs were seeded on transparent 96-well plates and cultured for 48 h in the presence of increasing concentrations of the BMS compounds or DMSO as a control (the concentration of DMSO was kept constant in all samples). Following the treatment, a metabolic activity test was performed with the use of Biolog Redox Dye Mix MB (BioLog), according to the manufacturer's instructions.

### PD1/PD-L1 checkpoint assay

The aAPCs were seeded in white 96-well plates at the density of 10 000 per well in the culture medium 24 h prior to the assay. On the day of the assay, 3.5x serial dilutions of the antibodies were prepared in the RPMI 1640-containing 1% FBS. Serial dilutions of BMS compounds were prepared in DMSO and formulated in RPMI 1640-containing 1% FBS. By this, the concentration of DMSO was kept constant in all samples. 95 μl of the medium was removed from the wells and the cells were overlaid with 40 μl of the compound dilutions. 20 000 of ECs were added to each well in 40 μl RPMI 1640 containing 1% FBS. Following 6 h incubation at 37°C, the plates were equilibrated at room temperature for 10 min and 80 μl of the Bio-Glo reagent (Promega) was added to each well. After incubation for 10 min, luminescence was quantified using FlexStation 3 (Molecular Devices). Half maximal effective concentrations (EC_50_) and maximal luminescence values (RLU_max_) were determined by fitting the Hill equation to the experimental data.

### PD-1/sPD-L1 effector assay

For the evaluation of the BMS impact on T cell inhibition by soluble PD-L1, the ECs were stimulated with the anti-CD3 antibody in the presence of the recombinant human sPD-L1. For this, the 96-well white flat bottom plates were coated overnight at 4°C with 5 μg/ml of the anti-CD3 antibody or the isotype control solution in PBS. The antibody solution was removed and the plates were washed 3 times with PBS and dried. sPD-L1 (aa 18–134) was diluted in PBS supplemented with the penicillin/streptomycin solution (100 U/ml final concentration each) in the presence of the BMS compounds or a corresponding volume of DMSO. Then, 15 μl of the solution was added to each well of the antibody-coated plate. ECs were centrifuged and diluted to 50 000 per ml, and 60 μl of the cell solution was added to each well. The final concentration of sPD-L1 was 10 μg/ml (0.6 μM). The final concentrations of the BMS compounds were: 0.12, 0.3, 1.2 and 3 μM, giving the following BMS:sPD-L1 molar ratios: 1:5, 1:2, 2:1 and 5:1. The cells were cultured for 24 h and the luciferase activity assay was performed using the Bio-Glo Luciferase Assay System (Promega) according to the manufacturer's instructions.

### Flow cytometry measurements

Binding of sPD-L1 (aa 18–134) to ECs was evaluated by flow cytometry. The His-tagged PD-L1 protein or its mutants were stained with NTA-Atto 647 N fluorescent dye (Sigma Aldrich) for 2 h at 22°C, at 8:1 molar ratio (protein:dye). PD-L1-Atto was formulated in 150 μl PBS with the tested compounds or antibodies. The samples were incubated for 30 min at 4°C in the dark. Meanwhile, ECs were centrifuged, washed with PBS and suspended in fresh PBS at concentration of 1 × 10^6^ cells per ml. 50 μl of ECs was added to each sample and incubated on ice for additional 60 min. The final concentrations of the components were: 25 μg/ml of PD-L1 (1.5 μM), 125 μg/ml the anti-PD-L1 antibodies and control antibodies and 1 μM of the BMS compounds. The samples were analyzed using the BD FACS Verse flow cytometer and BD FACSuite v1.0.6 software.

### Crystallization of the hPD-L1/BMS-1001 and hPD-L1/BMS-1166 complexes

Purified proteins in 10 mM Tris (pH 8.0) containing 20 mM NaCl, were concentrated to 5 mg/ml, mixed with the inhibitor in 1:3 molar ratio (protein:compound) and clarified by centrifugation at 15 000 × g for 10 min. Supernatant was used for screening using a sitting-drop vapor diffusion method. Diffraction-quality crystals were obtained at room temperature from 0.1 M Tris pH 8.5 containing 0.2 M magnesium chloride and 30% (w/v) PEG 4000 for the hPD-L1/BMS-1001 complex and from 0.01 M Tris pH 8.4 containing 0.28 M sodium chloride and 27% (w/v) PEG 4000 for hPD-L1/BMS-1166 complex.

### Structure solution and refinement

The X-ray diffraction data were collected at the PETRA III P11 beamline at DESY (Hamburg, Germany; [32]) and on the BL14.1 beamline operated by the Helmholtz-Zentrum Berlin (HZB) at the BESSY II (Berlin-Adlershof, Germany; [33]) for hPD-L1/BMS-1001 and hPD-L1/BMS-1166, respectively. The data were indexed and integrated using XDS [34, 35]. Scaling and merging was performed using Aimless [36]. Molecular replacement was calculated using Phaser [37] and PDB 5C3T as a search model. The model building was performed using Coot [38] and refinement was performed in Phenix [39]. Water molecules were added automatically and inspected manually Model validation was performed using Molprobity [40].

### Complex analysis by gel filtration

Superdex S75 10/30 HR column was equilibrated with 10 mM Tris pH 8.0 containing 20 mM NaCl. The column was calibrated with globular protein standards. The retention time of hPD-L1 (4 mg/ml) was determined. hPD-L1/inhibitor complexes were formed at 3:1 (inhibitor:protein) molar ratio, incubated for 15 min at room temperature and clarified by centrifugation for 10 min at 15 000 × g. The samples were analyzed in the same manner as the apo-hPD-L1.

### Cross-linking experiment

hPD-L1 was concentrated to 10 μM in PBS pH 7.4 and mixed with BMS-1001 or BMS-1166 in the 1:1 molar ratio, followed by the addition of the BS3 cross-linking reagent at 0.25, 0.5 or 1mM final concentration (Thermo Scientific). After 30-min incubation at room temperature, the reaction was quenched by the addition of Tris pH 7.5 to the final concentration of 25 mM. Control sample contained hPD-L1 and 1 mM BS3 reagent. The samples were analyzed by SDS-PAGE.

### Molecular docking

Docking of BMS compounds at the surface of PD-L1 was performed using Hex 8.0.0 with default parameters [41]. _A_PD-L1 and _B_PD-L1 protomers were extracted from co-crystal structures with BMS-1001 or −1166, described in this manuscript, and used as receptors. BMS compounds were also extracted from the co-crystal structures, served as ligands and treated as rigid.

### Accession numbers

Coordinates and structure factors were deposited in the Protein Data Bank with accession numbers 5NIU (BMS-1001) and 5NIX (BMS-1166).

## SUPPLEMENTARY MATERIALS FIGURES



## Abbreviations

TCR: T-cell receptor
aAPCs: artificial antigen-presenting cells
sPD-L1: soluble PD-L1
FDA: Food and Drug Administration
BMS: Bristol-Myers Squibb
ECs: PD-1 Effector Cells
AIDA: Antagonist Induced Dissociation Assay
HTRF: Homogenous Time-Resolved Fluorescence.
