# Supplementary material for: Small-molecule inhibitors of PD-1/PD-L1 immune checkpoint alleviate the PD-L1-induced exhaustion of T-cells
Source: Oncotarget. 2017 Aug 7;8(42):72167–81. doi: 10.18632/oncotarget.20050 (PMC5641120; doi:10.18632/oncotarget.20050)
Supplement: Supplementary file 1 [file oncotarget-08-72167-s001.pdf]

Small-molecule inhibitors of PD-1/PD-L1 immune checkpoint alleviate the PD-L1-induced exhaustion of T-cells

SUPPLEMENTARY MATERIALS

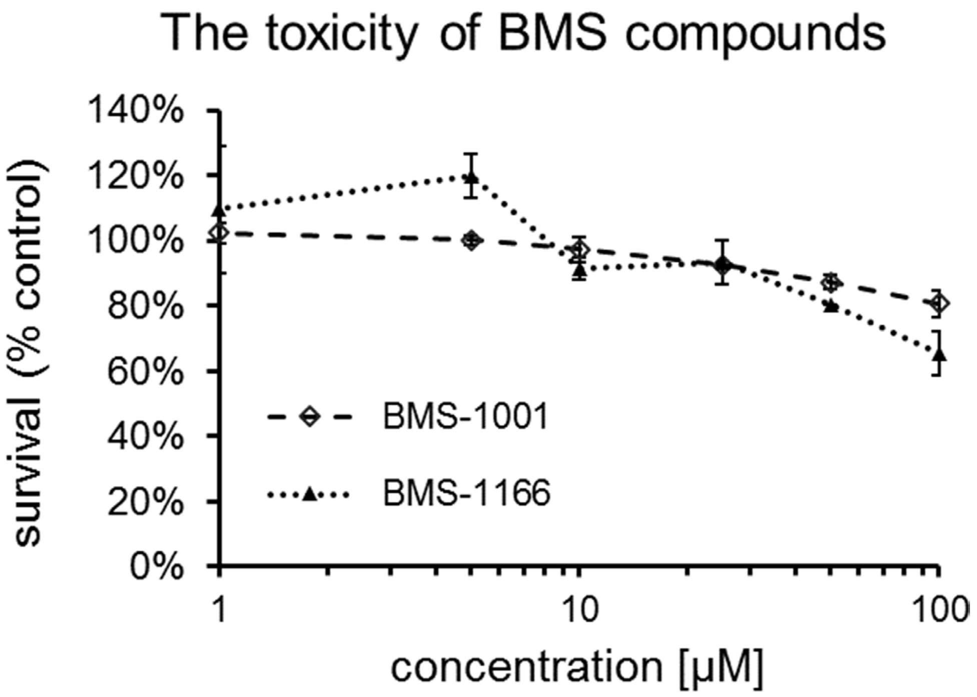

Supplementary Figure 1: The toxicity of BMS-1001 and -1166 towards aAPCs. The toxicity of BMS-1001 and -1166 compounds against the PD-L1 aAPC/CHO-K1 cells (aAPCs) was tested following the 48 h treatment with the indicated concentrations of the compounds. The graph shows the representative result from 3 experiments.

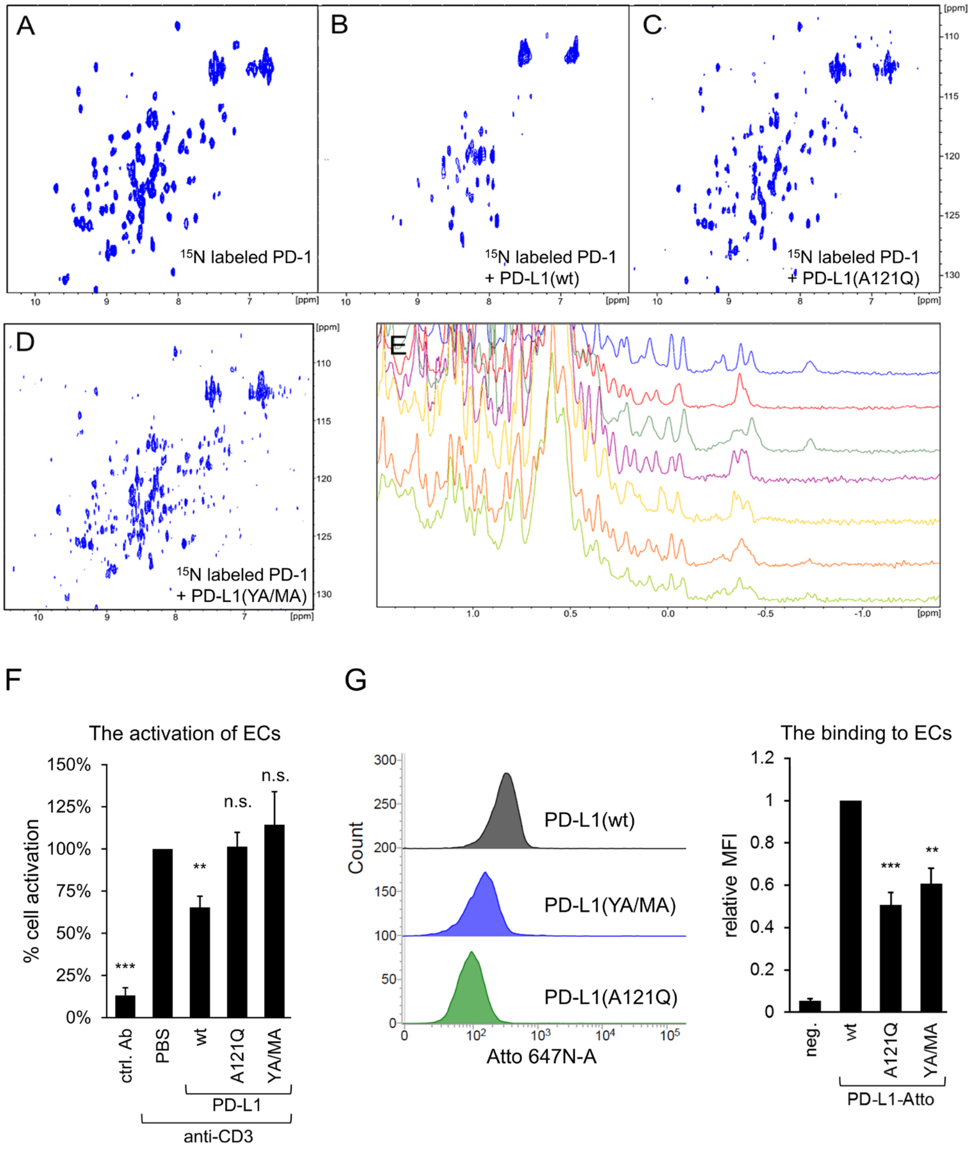

Supplementary Figure 2: The analysis of PD-L1(A121Q) and PD-L1(Y56A, M115A) in respect to PD-1 binding and activating potential of ECs. (**A**–**D**) ^{1}H-^{15}N HMQC spectra of the ^{15}N labeled hPD-1 alone (A), ^{15}N labeled hPD-1 and unlabeled hPD-L1(wt) (B), ^{15}N labeled hPD-1 and unlabeled PD-L1(A121Q) (C), and ^{15}N labeled hPD-1 and unlabeled PD-L1(Y56A, M115A), called PD-L1(YA/MA) (D). (B) Only a small number of cross-peaks are detected due to the increased relaxation associated with the formation of PD-1/PD-L1 complex. (C, D) No changes in chemical shifts of the ^{15}N labeled hPD-1 are observed upon the addition of slight excess of the mutant proteins, which indicates the lack of interaction of the proteins with PD-1. (**E**) The aliphatic regions of the ^{1}H NMR spectra are shown. At ca. d = 0.4 ppm the NMR signals from hPD-1 (blue) and wt-hPD-L1 (red) overlay and broaden in the spectrum of the pre-formed hPD-1/wt-hPD-L1 complex (green). In the spectra of hPD-1 and the A121Q mutant (purple), and YA/MA (yellow) only overlapping signals from hPD-1 (blue) and the A121Q mutant (orange), and YA/MA (light green) are observed. (**F**) ECs were activated for 24 h with the anti-CD3 antibody alone or in the presence of sPD-L1(wt) or the indicated mutants. The activity of luciferase was determined as an indicator of cell activation. The graphs present mean ± SEM from at least three independent experiments. Statistical significance was evaluated using a one-way ANOVA with the Tukey's post-hoc test: $**p < 0.01$, $***p < 0.001$. (**G**) The binding of the fluorescently-labeled PD-L1(wt), PD-L1(A121Q) or PD-L1(YA/MA) to aAPCs determined using flow cytometry. MFI – relative Geo Mean Fluorescence Intensity values. The bar graphs present mean ± SEM from three independent experiments. For the statistics, *t*-test was used: $**p < 0.01$, $***p < 0.001$.

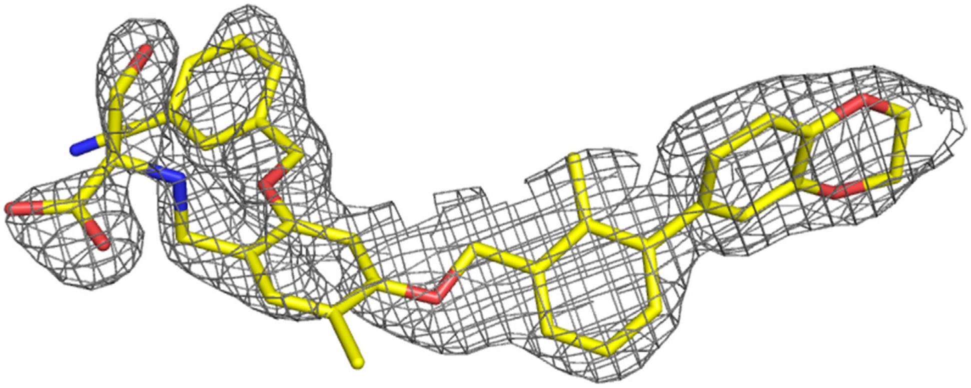

Supplementary Figure 3: Quality of electron density map for BMS-1001 molecule located inside the PD-L1 dimer. Example of the 2Fo-Fc omit map contoured at 1σ shows the well-defined density for the BMS-1001 compound molecule.

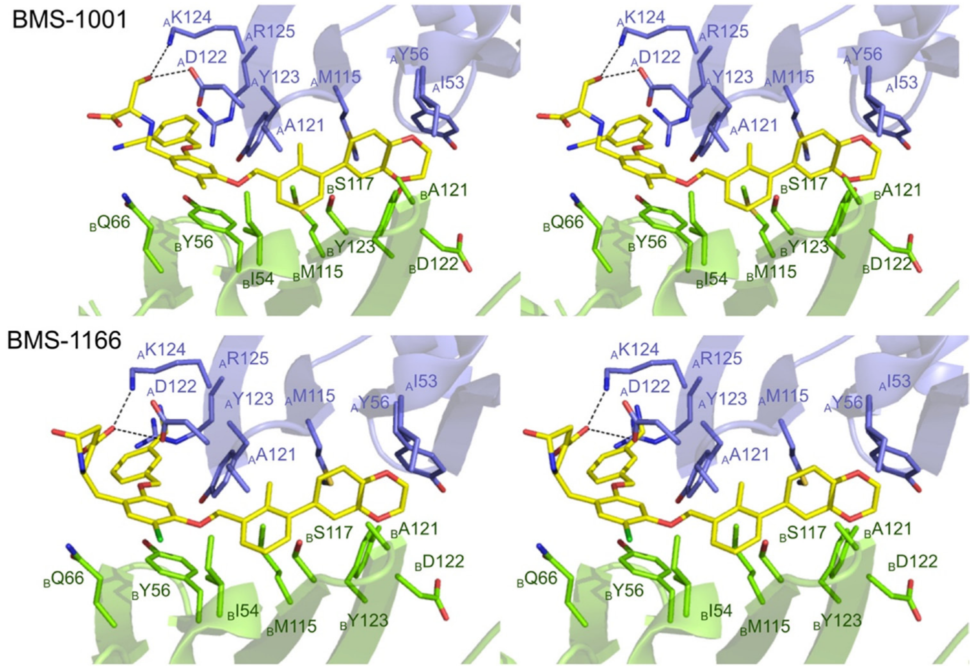

Supplementary Figure 4: Detailed interactions of BMS-1001 and BMS-1166 with PD-L1 molecules. Both inhibitors show similar binding modes within the 2,3-dihydro-1,4-benzodioxine moiety and central ring creating multiple hydrophobic interactions with both PD-L1 protomers. Flexible tails of both inhibitors create hydrogen bonds with lysine124 and aspartate122 stabilizing the 2,3-dihydro-1,4-benzodioxine group outside the binding tunnel.

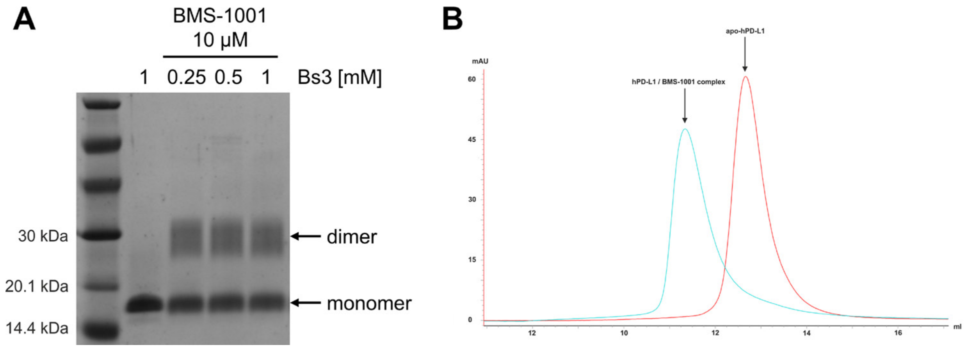

Supplementary Figure 5: BMS-1001 and BMS-1166 provoke PD-L1 dimerization in solution. (**A**) Cross-linking of hPD-L1 in presence of the BMS-1001 compound. Bands corresponding to the hPD-L1 monomer and dimer after cross-linking are present - indicating BMS-1001 induced dimerization. Control sample (hPD-L1 with 1 mM BS3 in absence of BMS-1001) contains only one band corresponding to molecular weight of the monomer. (**B**) Size exclusion chromatograms of hPD-L1 in the presence and absence of BMS-1001. The Apo-hPD-L1 peak exhibits longer retention time compared to that of the hPD-L1/BMS-1001 complex indicating the lower molecular weight corresponding to the monomer size. The peak for the hPD-L1/BMS-1001 complex is shifted and corresponds to the molecule with a double size of the monomer (the hPD-L1 dimer).
